# Age-Related Association of Refractive Error with Intraocular Pressure in the Korea National Health and Nutrition Examination Survey

**DOI:** 10.1371/journal.pone.0111879

**Published:** 2014-11-04

**Authors:** Jin A. Choi, Kyungdo Han, Yong-Moon Park, Chan Kee Park

**Affiliations:** 1 St. Vincent's Hospital, Department of Ophthalmology, College of Medicine, the Catholic University of Korea, Suwon, Korea; 2 Department of Biostatistics, College of Medicine, the Catholic University of Korea, Seoul, Korea; 3 Department of Preventive Medicine, College of Medicine, the Catholic University of Korea, Seoul, Korea; 4 Department of Epidemiology and Biostatistics, Arnold School of Public Health, University of South Carolina, Columbia, South Carolina, United States of America; 5 Seoul St. Mary's Hospital, Department of Ophthalmology, College of Medicine, Catholic University of Korea, Seoul, Korea; Medical College of Soochow University, China

## Abstract

**Background:**

To investigate the distribution of intraocular pressure (IOP) and refractive errors according to age group in a representative sample of non-glaucomatous Korean adults.

**Methods:**

A total of 7,277 adults (≥19 years) who participated in the Korea National Health and Nutrition Examination Survey (KNHANES) from 2008 to 2011 underwent ophthalmic examination were divided into three groups according to age: the young- (19–39 years), middle- (40–59 years), and old- (≥60 years) age groups. Simple and multiple regression analyses between IOP and various parameters (including the refractive error) were conducted.

**Results:**

The mean IOP of the total population was 14.0±0.1 mmHg [young: 13.9±0.1 mmHg; middle: 14.1±0.1 mmHg; old: 13.8±0.2 mmHg (*P* for trend = 0.085)]. Myopia and high myopia were more prevalent in the young- (70.8% and 16.1%, respectively), compared to the middle- (44.6% and 10.9%) and old- (8.9% and 2.2%) age groups. Univariate analysis in the total population showed that higher IOP was associated with myopic refractive error, the female gender, higher body mass index (BMI), diabetes, hypertension, and hypercholesterolemia (all *P*<0.05). In the young- and middle-age groups, higher IOP was associated with myopic refractive error, the female gender, higher BMI, hypercholesterolemia and diabetes (all *P*<0.05). In the old-age group, the association between IOP and refractive error was not significant (*P* = 0.828). In multiple linear regression analysis, similar significant relationships between the refractive error and IOP were found in the young- and middle-age groups (beta = −0.08 and −0.12; *P* = 0.002 and <0.001 for young- and middle-age group, respectively), but not in the old-age group (beta = 0.03; *P* = 0.728), after adjusting for age, gender, BMI, region of habitation, diabetes, hypertension, and hypercholesterolemia.

**Conclusions:**

Myopic refractive error was an independent predictor of higher IOP in non- glaucomatous eyes, and the association between refractive error and IOP differed according to age.

## Introduction

Elevated intraocular pressure (IOP) is the most important modifiable risk factor for glaucoma [1]. Although the prevalence of glaucoma is known to increase with age in adults [2], the association between age and IOP remains controversial. While many previous studies in Western populations demonstrated a positive correlation between age and IOP [3], [4], several studies conducted in Korean, Chinese and Japanese populations revealed a negative or no correlation between age and IOP [5]–[8]. In one longitudinal study in a Japanese population, however, the IOP decreased significantly with age [9]. These inconsistent results among different ethnic populations suggest that different characteristics, which vary among ethnicities, may play a role in determining IOP according to age.

Many studies have assessed the risk factors of elevated IOP. Systolic and diastolic blood pressure (BP), hypertension, diabetes, hypercholesterolemia, body mass index (BMI) and waist circumference—most of which are age-related factors—show positive associations with IOP [10]–[14].

Regarding refractive error, a negative association with IOP has been reported in several studies [10], [15], [16]. Myopia has emerged as a major health issue worldwide, and its prevalence has increased rapidly in the past few decades, particularly in East Asia [17]–[21]. In a study reporting the prevalence of major eye diseases in Korea, the prevalence of myopia was greatest in those aged 12–18 years (78.8%), followed by those aged 19–29 years (75.3%) [22]. In a recent study involving 19-year-old males in Korea, the prevalence of myopia was higher, at 96.5% [18]. Scleral thinning associated with myopia may reduce the tolerance of the sclera and optic nerve to elevated IOP, thereby inducing to further axial elongation of the sclera and injuries to optic nerve head. In addition, sclera becomes stiffer with aging, subject to higher stresses at elevated IOP [23]. In this regard, altered scleral tissue characteristics associated with myopia and aging may contribute to the negative association between age and IOP in these populations. However, little is known of the age-related association between myopia and IOP. Therefore, we investigated the distribution of IOP and refractive errors according to age group in a representative sample of non-glaucomatous Korean adults.

## Patients and Methods

### Study population

The Korea National Health and Nutrition Examination Survey (KNHANES) is a nationwide, population-based and cross-sectional health examination and survey conducted regularly by the Division of Chronic Disease Surveillance, Korea Centers for Disease Control and Prevention, Ministry of Health and Welfare, to monitor the general health and nutritional status of people in South Korea. To date, KNHANES has been performed in 1998 (KNHANES I), 2001 (KNHANES II), 2005 (KNHANES III), 2007–2009 (KNHANES IV) and 2010–2012 (KNHANES V). It consists of a health interview survey, a nutrition survey and a health examination survey. A stratified, multistage probability sampling design is used for the selection of household units that participate in the survey. Additional details regarding the study design and methods are provided elsewhere [10], [15], [18].

Data from the fourth (KNHANES IV-2&3, 2008, 2009) and fifth (KHANES V 1&2, 2010, 2011) KHANES were used to estimate the association between the refractive error and IOP. The mean age of the study population was 38.3±0.2 years (range, 3–97 years). Among 15,932 participants, 12,496 individuals aged ≥19 years were selected for the current study. Among these, 12,496 individuals who met the following inclusion criteria: received the ophthalmologic investigation of the survey; did not have glaucoma or suspicion of glaucoma (described below); were not pseudophakic or aphakic; had no history of cataract, refractive, retinal or strabismus surgery; did not have pterygium. The standardized Lens Opacities Classification System (LOCS) III photographic images were used to assess cataracts. Cataract was defined as nuclear (LOCS III score ≥4 for nuclear opalescence or nuclear color), cortical (LOCS III score ≥2 for cortical cataracts), posterior subcapsular (PSC, LOCS III score ≥2 for PSC), and mixed cataracts (more than one type per eye) compared with standard photographs. Based on this definition, participant with cataracts were excluded. KNHANES IV and V were conducted according to the Declaration of Helsinki. All participants in the survey signed an informed consent form. The Institutional Review Board of St. Vincent Hospital, College of Medicine, the Catholic University of Korea, Seoul, Korea approved the protocol (local IRB approval number: VC13RISI0120). Patient records/information was anonymized and de-identified prior to analysis.

### Assessment of refractive status

As part of the standardized ophthalmic examination, all participants underwent autorefraction in both eyes using a non-accommodative picture target with standard background illumination on the Topcon KR8800 autorefractor (Topcon, Tokyo, Japan). The spherical equivalent (SE) refractive error was calculated as sphere +1/2 cylinder. Myopia was defined by an SE ≤−0.50 diopters (D); mild myopia was defined as >−3.0 D; moderate myopia was defined as ≤−3.0D; and high myopia was defined as ≤−6.0D.

### Examination of baseline IOP and glaucoma

Intraocular pressure was measured once per each eye from right to left, by a trained ophthalmologist with a Goldmann applanation tonometer (Haag-Streit model BQ-900; Haag-Streit AG, Koeniz, Switzerland) prior to perimetry and fundus photography.

Digital fundus images were obtained using a digital non-mydriatic retinal camera (TRC-NW6S; Topcon, Inc., Tokyo, Japan) and a Nikon D-80 digital camera (Nikon, Inc., Tokyo, Japan). Based on the digital fundus images, the vertical cup-to-disc ratios (VCDRs) were measured. For participants meeting the glaucoma suspicion criteria (having elevated IOP ≥22 mmHg or a glaucomatous optic disc), visual-field testing was performed with frequency doubling technology (FDT) (Humphry Matrix Carl Zeiss Meditec, Inc., Dublin, CA) using the N30-1 screening test. We excluded eyes with glaucoma (defined as follows) or a history of glaucoma surgery. Use of glaucoma medication was not considered as an exclusion criteria. Participants were defined as having primary open angle glaucoma based on the criteria of the International Society of Geographical and Epidemiological Ophthalmology criteria as follows: [24] Category 1 required both a VF defect consistent with glaucoma and either a VCDR ≥0.7 or the difference of the VCDR between both eyes of ≥0.2; category 2 (when the VF test was not reliable or available) required VCDR ≥0.9 or the difference of the VCDR between both eyes of ≥0.3; and category 3 (when no VF testing or optic disc examination were available) required a visual acuity of ≤20/400 and the IOP greater than 21 mmHg.

### Other variables

Demographic variables included age, gender, urban or rural region of residence, income and education level. Among the 16 districts of South Korea, eight major cities (Seoul, Gyeonggi, Busan, Daegu, Incheon, Gwangju, Daejeoun and Ulsan) were grouped as urban areas, and the other provinces (Gangwon, Chungbuk, Chungnam, Jeonbuk, Jeonnam, Gyeongbuk, Gyeongnam and Jeju) were grouped as rural areas. Participants were categorized in the low-income group if their income belonged to the lowest quartile.

A specially trained examiner recorded the anthropometric measurements. The BP was measured in the right arm using a standard mercury sphygmomanometer (Baumanometer, WA Baum Co., New York, USA) after 5 min of rest in the sitting position. Systolic and diastolic BPs were measured three times at 30-s intervals; the second and third measurements were then averaged to calculate the final BP. Hypertension was defined as a systolic hypertension ≥140 mmHg or diastolic pressure ≥90 mmHg, or the use of antihypertensive medication. Diabetes was defined as having fasting glucose concentration ≥126 mg/dL, or taking prescribed insulin or oral anti-diabetic medication. Hypercholesterolemia was defined as total cholesterol (TC) ≥240 mg/dL or treatment for high TC. Height was measured to the nearest 0.1 cm using a portable stadiometer (SECA 225, SECA Deutschland, Hamburg, Germany) when participants were standing barefoot. Body weight was measured to the nearest 0.1 kg on a balanced scale (GL-6000-20, CAS KOREA, Seoul, Korea) while participants wore a lightweight gown. BMI was calculated as the individual's weight in kilograms divided by the square of their height in meters. Waist circumference was measured to the nearest 0.1 cm in a horizontal plane at the level of the midpoint between the iliac crest and the costal margin at the end of normal expiration.

### Statistical analyses

Statistical analyses were performed using the SAS survey procedure (version 9.2; SAS Institute, Inc., Cary, NC, USA) to reflect the complex sampling design and sampling weights of KNHANES and to provide representative national prevalence estimates. The procedures included unequal probabilities of selection, oversampling and non-response so that inferences could be made about the Korean adult participants.

Participants were divided into three groups according to age (19–39, 40–59, and ≥60 years), and variables were characterized according to age using means and standard errors for continuous variables and numbers and percentages for categorical variables. The prevalence of myopia and high myopia in each age group was determined.

Simple and multiple linear regression analyses between IOP and various parameters (including the refractive error) were assessed in the total study population and each age group. For multiple linear regression analysis, we first adjusted for age and gender (Model 1). Next, we adjusted for age, gender and other confounders such as BMI, area of residence, diabetes, hypertension, and hypercholesterolemia (Model 2). For all analyses, *P* values were two-tailed and <0.05 was considered to indicate statistical significance.

## Results

Among 12,496 participants aged 19 year or more, 5,219 were excluded; 165 had glaucoma, 1,262 had prior cataract, strabismus, retinal or refractive surgery, 3,663 had cataract or pteryium, and 129 had missing values. Finally, a total of 7,277 adult non-glaucomatous participants (3,098 males and 4,179 females) were included in this analysis.

Mean values of systemic, ocular and demographic characteristics by age group are shown in Table 1. Because the IOPs in the right and left eyes were highly correlated (Pearson's correlation = 0.86, *P*<0.001), data for only the right eyes were used.

**Table 1:** Characteristics of the Study Participants.

		Young-age group	Middle-age group	Old-age group	
	Total study population	19–39 yrs	40–59 yrs	≥60 yrs	P value
*n*	7,277	3,343	3,303	631	
Systemic and ocular characteristics					
Age, years	38.9±0.2	29.6±0.2	47.9±0.1	64.8±0.2	<0.001
Gender, % females	50.9±1.8	47.1±1.0	48.9±0.8	45.6±2.2	<0.001
Height, cm	165.5±0.1	167.7±0.2	163.4±0.2	159.7±0.4	<0.001
Weight, kg	64.9±0.2	65.4±0.3	64.5±0.2	62.0±0.4	<0.001
Waist circumference, cm	80.5±0.3	78.9±0.5	82.0±0.2	84.7±0.4	<0.001
BMI, kg/m^2^	23.6±0.1	23.1±0.1	24.1±0.1	24.3±0.1	<0.001
Diabetes, %		2.1±0.3	8.2±0.6	14.7±1.8	<0.001
Hypertension, %		9.3±0.6	27.4±1.0	58.9±2.7	<0.001
Systolic BP, mmHg	115.3±0.2	111.0±0.3	119.3±0.4	129.0±1.0	<0.001
Diastolic BP, mmHg	76.5±0.2	74.0±0.2	79.4±0.3	77.9±0.6	<0.001
Hypercholesterolemia	9.5±0.4	4.8±0.4	13.7±0.7	26.5±2.5	<0.001
Refractive error, Diopter	−1.3±0.0	−1.9±0.1	−1.1±0.0	0.6±0.1	<0.001
Intraocular pressure, mmHg	14.0±0.1	13.9±0.1	14.1±0.1	13.8±0.2	0.096
Demographic characteristics					
Area of residence, % of Rural regions	20.4±3.2	13.5±1.7	19.6±2.3	24.8±4.0	<0.001
Education level, % of 9 yrs or more	63.7±1.8	96.9±0.4	69.1±2.3	24.8±4.0	<0.001
Income, % of lowest quartile	21.8±1.8	9.2±0.8	19.6±2.3	24.8±4.0	<0.001

BMI = body mass index; IOP = intraocular pressure; BP = blood pressure. Values are means ± standard deviation.

**Figure 1:**
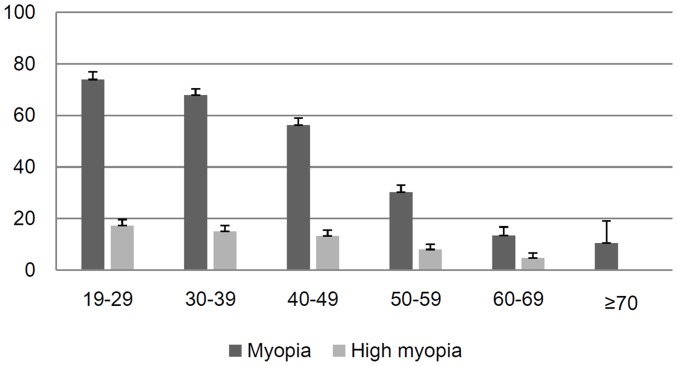
Prevalence of myopia (−0.5 D or greater myopia) and high myopia (−6.0 D or greater myopia) in different age groups.

The prevalence of myopia and high myopia according to age groups are shown in Figure 1. The prevalence of myopia and high myopia was dramatically higher in the young- (70.8% and 16.1%, respectively), compared to the middle- (44.6% and 10.9%) and old-age groups (8.9% and 2.2%). The mean IOP of the sample was 14.0±0.1 mmHg [(13.9±0.1 mmHg in the young-, 14.1±0.1 mmHg in the middle-, and 13.8±0.2 mmHg in the old-age group (*P* for trend = 0.085)].

**Figure 2:**
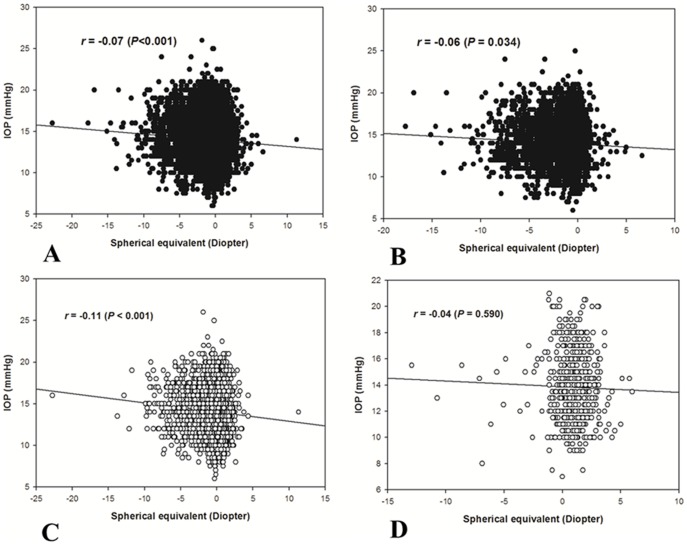
Association between intraocular pressure and refractive error in the total study population (A) and young (B), middle (C) and old age group (D).

Figure 2 shows the IOP distribution according to the refractive error (SE) in the total study population and young, middle and old age groups. A negative relationship between IOP and SE was detected after adjustment for age (r = −0.07, *P*<0.001) in total population (Fig. 2A). IOP and SE showed a negative relationships in young and middle age group (r = −0.06, *P* = 0.034 in young age group shown in Fig. 2B; r = −0.11, *P*<0.001 in middle age group shown in Fig. 2C), whereas no significant relationship between IOP and SE was noted in old age group (r = −0.04, *P* = 0.590 shown in Fig. 2D).

Univariate analysis in the total study population showed that higher IOP was associated with myopic refractive error, female gender, higher BMI, diabetes and hypertension (all *P*<0.05; Table 2). However, the correlations of IOP with various parameters differed according to age group (Table 2). In the young- and middle-age groups, higher IOP was associated with myopic refractive error, female gender, higher BMI, and diabetes (all *P*<0.05). In the old-age group, higher IOP was associated only with BMI (*P*<0.001), while the association between IOP and refractive error was not identified (*P* = 0.828).

**Table 2:** Univariate Associations of Intraocular Pressure with Various Parameters including Refractive Error in Total Population and Young, Middle and Old Age Groups.

	Total population			Young-age group			Middle-age group			Old-age group		
	Beta	SE	*P*	Beta	SE	*P*	Beta	SE	*P*	Beta	SE	*P*
Refractive error	−0.06	0.02	0.001	−0.06	0.02	0.034	−0.12	0.03	<0.001	−0.02	0.08	0.828
Age	0.01	0.00	0.123	0.03	0.01	0.002	−0.01	0.01	0.363	−0.01	0.03	0.763
Gender	−0.32	0.07	<0.001	−0.31	0.11	0.003	−0.43	0.10	<0.001	0.60	0.25	0.015
BMI	0.07	0.01	<0.001	0.07	0.02	0.000	0.08	0.02	<0.001	0.14	0.04	<0.001
Region of habitation	−0.10	0.18	0.573	−0.25	0.27	0.354	0.00	0.18	0.996	0.25	0.35	0.472
Diabetes	0.51	0.18	0.004	1.07	0.42	0.011	0.47	0.22	0.034	−0.01	0.36	0.981
Hypertension	0.25	0.11	0.020	0.46	0.20	0.022	0.22	0.14	0.113	0.31	0.29	0.286
Hypercholesterolemia	0.47	0.13	<0.001	0.70	0.22	0.001	0.34	0.18	0.058	0.56	0.33	0.095
Education level	−0.07	0.11	0.545	−0.29	0.36	0.431	0.02	0.13	0.866	−0.32	0.26	0.217
Income status	−0.06	0.14	0.646	0.06	0.23	0.811	−0.17	0.20	0.402	−0.03	0.26	0.892

SE = standard error; BMI = body mass index.

Multiple linear regression analysis revealed that IOP was significantly associated with refractive error after adjustment for age, gender, BMI, area of residence, diabetes, and hypertension (*P*<0.001) in the total study population (Table 3). A significant relationship between the refractive error and IOP was found in the young- and middle-age groups (*P* = 0.002 and <0.001, respectively), but not in the old-age group (*P* = 0.728). BMI was significantly associated with higher IOP in the old-age group by multiple linear regression analysis (*P* = 0.004). The associations between refractive error and IOP in sub-population by decades are shown in the table S1.

**Table 3:** Association between Refractive Error (D) and Intraocular Pressure in Total Studied Population, Young, Middle, and Old age Group.

	Model 1: adjusted for age and gender			Model 2: adjusted for age, gender, BMI, area of residence, diabetes, hypertension, and hypercholesterolemia		
	Beta	SE	P value	Beta	SE	P value
Total population						
Refractive Error	−0.09	0.02	<0.001	−0.10	0.02	<0.001
Young age group						
Refractive Error	−0.07	0.02	0.006	−0.08	0.03	0.002
Middle age group						
Refractive Error	−0.10	0.02	0.005	−0.12	0.03	0.001
Old age group						
Refractive Error	−0.06	0.12	0.626	0.03	0.08	0.728

SE = standard error; BMI = body mass index.

## Discussion

In this study, we found a significant association between refractive error and IOP in non-glaucomatous Korean adults aged ≥19 years. Myopic refractive error appears to be an independent predictor of high IOP after adjusting for potential confounding factors, such as age, gender, BMI, region of habitation, diabetes, and hypertension (Table 3). Our results are in agreement with the Beaver Dam study and other previous studies of the association between myopia and IOP [10], [16].

To address the inconsistent association between age and IOP according to ethnic group, we focused on the rapid myopic shift observed in young East Asians. In accordance with recent reports [20], the prevalence of myopia and high myopia was increased dramatically in the young subjects (Fig. 1). Interestingly, the association between refractive error and IOP differed according to age group in this study. A significant relationship was detected between myopia refractive error and IOP in the young- and middle-age groups; only BMI was associated with IOP in the old-age group (Table 2). In accordance with previous reports [7], [13], Table 1 shows that diabetes, waist circumference, BMI, prevalence of diabetes and hypertension—which are known to be associated with higher IOP—are increased with age. Our results suggested that the influences of risk factors on IOP differ according to age. Myopia appears to exert a marked influence on IOP in young-to-middle-age subjects, whereas age-related risk factors such as BMI exert a greater influence on IOP in old-age subjects. In accordance with our study, Park et al. reported that IOP was associated with metabolic syndrome in postmenopausal, but not premenopausal, non-glaucomatous Korean adult females [25]. It was speculated that the sympathetic activity stimulated by increased insulin resistance and decreased estrogen action on the inflow and outflow facility was associated with the elevated IOP in older populations (postmenopausal females).

The mechanism underlying the myopia and elevated IOP is largely unknown. One possible reason is associated with the differences in tissue biomechanics according to age. In the human posterior sclera, there are regional variations in the mechanical strains, and the posterior sclera in particular is subjected to higher tensile strain compared to the adjacent mid-peripheral sclera [26]. In normal posterior sclera specimens, older specimens were associated with lower tensile strain, while younger specimens exhibited greater tensile strains and lower fiber stiffness [27]. With respect to the age of onset, the majority of myopia cases occur in subjects less than 20 years of age, although adult-onset (20 year or older) myopia also exists [28]. In this regard, the IOP may exert some degree of influence on the axial elongation of the posterior sclera, particularly in younger compared to older subjects, even in the adult population. The different age-related associations between refractive error and IOP in our study may support this hypothesis. In a recent report, more severe myopia was associated with a greater likelihood of glaucoma in a Korean population aged 40 years or more [15]. This positive correlation between refractive error and IOP emphasizes the importance of glaucoma surveillance in the myopic population because both elevated IOP and myopia are important risk factors for glaucoma development [16].

In Namil study, the first population based study on Korean adults 40 and above, IOP tended to decrease by approximately 0.2 mmHg when age increased by 10 years [8]. However, we found no significant association between age and IOP, in accordance with another Korean population based study by Kim et al. [10]. The possible reason for the inconsistent results between studies is that the inclusion criteria and the adjusting factors are different between studies. In this study, we excluded eyes with glaucoma, and adjusted the BMI, presence of hypertension and diabetes, which are increased with aging, which may have influenced on the final results. In addition, the two former studies reported that refractive error was significantly associated with IOP, which are in consistent with our study. However, the analysis in young generation (<40 years or age) and age-stratified analysis were not performed in the former studies.

Although the strengths of this study—including inclusion of a large, nationally representative population—enabled detection of an age-stratified association between IOP and various parameters, this study had several limitations. Because of its cross-sectional design, the association between SE and IOP may not imply a causal relationship, and so a longitudinal study is needed to confirm our finding. This study may be confounded by a lack of data on relevant variables such as central corneal thickness, curvature and axial length. The Goldmann applanation tonometry used in this study is the gold standard for measuring IOP [21], [29]. However, this is affected by central corneal thickness, albeit to a lesser extent than noncontact pneumotonometry. Finally, the refractive status was not checked under cycloplegic conditions and the range of hyperopic refractive errors could be compressed in the regression analysis due to involuntary accommodation. However, children and adolescents whose refractions are more likely affected by accommodation were excluded from this study. These limitations should be addressed in future work.

In summary, a positive association between the myopic refractive error and elevated IOP was confirmed in a large Korean adult population. Additionally, we found that the association between refractive error and IOP differs according to age. These findings suggest that approaches specific to age groups should be adopted in the screening and clinical diagnosis of glaucoma, for which elevated IOP is the most important risk factor.

## Supporting Information

Table S1 Association between Refractive Error (D) and Intraocular Pressure in Sub-Population by Decades.
(DOCX)
