# Supplementary material for: Age-Related Association of Refractive Error with Intraocular Pressure in the Korea National Health and Nutrition Examination Survey
Source: PLoS One. 2014 Nov 4;9(11):e111879. doi: 10.1371/journal.pone.0111879 (PMC4219793; doi:10.1371/journal.pone.0111879)
Supplement: Table S1 — Association between Refractive Error (D) and Intraocular Pressure in Sub-Population by Decades. (DOCX) [file pone.0111879.s001.docx]

**Table S1. Association between Refractive Error (D) and Intraocular Pressure in Sub-Population by Decades**

|  | **Model** | | |
| --- | --- | --- | --- |
|  | **: adjusted for age, gender, BMI, area of residence, diabetes, hypertension, and hypercholesterolemia** | | |
|  | **Beta** | **SE** | **P value** |
| 20-29 |  |  |  |
| Refractive Error | -0.10 | 0.04 | 0.014 |
| 30-39 |  |  |  |
| Refractive Error | -0.06 | 0.03 | 0.039 |
| 40-49 |  |  |  |
| Refractive Error | -0.10 | 0.04 | 0.005 |
| 50-59 |  |  |  |
| Refractive Error | -0.17 | 0.05 | <0.001 |
| 60-69 |  |  |  |
| Refractive Error | -0.01 | 0.08 | 0.863 |
| 70- |  |  |  |
| Refractive Error | 0.28 | 0.27 | 0.292 |

SE = standard error; BMI = body mass index
